# Effect of Experiential Vegetable Education Program on Mediating Factors of Vegetable Consumption in Australian Primary School Students: A Cluster-Randomized Controlled Trial

**DOI:** 10.3390/nu12082343

**Published:** 2020-08-05

**Authors:** Astrid A. M. Poelman, Maeva Cochet-Broch, Bonnie Wiggins, Rod McCrea, Jessica E. Heffernan, Janne Beelen, David N. Cox

**Affiliations:** 1 Sensory and Consumer Science, CSIRO Agriculture and Food, North Ryde, NSW 2113, Australia; maeva.broch@csiro.au (M.C.-B.); jess.heffernan@csiro.au (J.E.H.); janne.beelen@csiro.au (J.B.); 2 Public Health Nutrition, CSIRO Health and Biosecurity, Adelaide, SA 5000, Australia; bonnie.wiggins@csiro.au (B.W.); david.cox@csiro.au (D.N.C.); 3 Adaptive Communities and Industries Group, CSIRO Land and Water, Dutton Park, QLD 4102, Australia; rod.mccrea@csiro.au

**Keywords:** children, primary (elementary) school, sensory, vegetable, acceptance, experiential learning, education program, cluster-randomized controlled trial (cluster-RCT)

## Abstract

Schools provide a relevant and equitable environment to influence students towards increased vegetable consumption. This study aimed to evaluate the effectiveness of a Vegetable Education Resource To Increase Children’s Acceptance and Liking (VERTICAL) for Australian primary schools (curriculum aligned and based on a framework of food preference development and sensory experiential learning) on positively influencing factors predisposing children towards increased vegetable consumption. The secondary aim was to evaluate two levels of teacher training intensity on intervention effectiveness. A cluster-RCT amongst schools with three conditions was conducted: 1 = teaching VERTICAL preceded by online teacher training; 2 = as per 1 with additional face-to-face teacher training; 3 = Control. Pre-test, post-test and 3-month follow-up measures (knowledge, verbalization ability, vegetable acceptance, behavioural intentions, willing to taste, new vegetables consumed) were collected from students (*n* = 1639 from 25 schools in Sydney/Adelaide, Australia). Data were analyzed using mixed model analysis. No difference in intervention effectiveness was found between the two training methods. Compared to the Control, VERTICAL positively affected all outcome measures after intervention (*p* < 0.01) with knowledge sustained at 3-month follow-up (*p* < 0.001). In conclusion, VERTICAL was effective in achieving change amongst students in mediating factors known to be positively associated with vegetable consumption.

## 1. Introduction

Less than 4% of Australian primary school-aged children meet vegetable intake recommendations [1] and in the USA, 4–8 year olds consume, on average, less than a cup of vegetables per day [2]. Vegetable consumption is important for prevention of cardiovascular diseases and certain types of cancers [3]. Low acceptance is a key barrier to vegetable consumption amongst children and vegetables are the food category least liked by children [4,5]. In contrast to other core food groups, vegetables do not have sensory properties that are innately liked and can be more intense in an innately disliked taste, bitterness [6]. Most of our food preferences are learned [7,8,9,10], and thus, in particular, the acceptance of the sensory properties of vegetables needs to be acquired.

A recent cross-cultural study amongst 14 countries from five continents showed that people in three countries of traditional Anglo-Saxon background/heritage (UK, USA and Australia) did not associate ‘fruit and vegetables’ with ‘feeling good’ as often as people from many other countries, such as France, Italy and Norway [11]. Moreover, where French consumers implicitly associated healthy with tasty [12], the opposite was the case for US-Americans [13]. Thus, in particular countries of Anglo-Saxon heritage may require the development of a more positive attitude towards eating vegetables to contribute to closing the gap between current and recommended intakes.

Schools provide equitable access for children to learn about healthy eating in general and vegetables specifically, and offer an environment to adopt more positive attitudes and preferences. School settings are independent of parental knowledge, habits, attitudes, time and economic means, and negative parenting styles, which may all pose barriers to adoption of healthy eating habits [14,15,16,17]. They provide a way to break negative family interplay [16] and provide opportunities for positive reinforcement from teachers and peers [18].

School-based nutrition interventions rarely focus on vegetables exclusively, rather predominantly target intake of fruit and vegetables simultaneously [19]. However, meta-analysis shows that such interventions merely benefit fruit intake, with an average increase of 0.24 portions of fruit in primary school-aged children but only 0.07 portions of vegetables [19]. Thus, specific vegetable targeted interventions are needed.

A systematic review and meta-analysis of primary school healthy eating programs showed that experiential learning strategies were associated with the largest effects in fruit and vegetable preference and consumption, with cross-curricular approaches and contingent reinforcement also proving successful strategies [20]; however, few school nutrition education programs include these elements [21]. In addition, none of the programs studied were vegetable-specific. These three elements of successful programs were all incorporated in a newly developed vegetable-specific education program for Australian primary schools aiming to positively predispose children to consuming vegetables, VERTICAL (Vegetable Education Resource to Increase Children’s Acceptance and Liking). The scientific framework of this short (5 h) teacher-led, curriculum-aligned experiential learning program is based on two main components: (1) scientific insights on children’s development of vegetable acceptance, including taste exposure, building familiarity and role modelling [8,9,22,23,24,25] and (2) sensory experiential learning elements from sensory education programs to ingest and describe vegetables using all senses [26,27,28,29,30,31,32].

A prototype version of VERTICAL was developed and showed positive changes in mediating factors associated with children’s vegetable consumption in a matched-control schools pilot study [33,34]. These changes included: increases in knowledge about vegetables and the senses, students’ verbalization skills around vegetable sensory properties, vegetable acceptance and willingness to try vegetables [34]. The program was evaluated favourably by teachers; however, preparation effort was seen as considerable [33]. The content of the VERTICAL program was refined based on study outcomes. In addition, an online teacher training module was developed to prepare teachers to teach the program.

This current study aimed to evaluate: (1) large-scale effectiveness of a vegetable education program on student outcomes (mediating factors associated with vegetable consumption) using a cluster-randomized controlled trial (cluster-RCT) and (2) effectiveness of two intensity levels of teacher training (low versus high) preceding the vegetable education program on these student outcomes. The research questions were the following:

- Does the vegetable education program improve primary school students’ knowledge, verbalization ability, vegetable acceptance, behavioural intentions, willingness to try vegetables and number of new vegetables consumed?
- Does a high-intensity training of teachers result in a greater effect of the vegetable education program than a low-intensity training of teachers?

We hypothesized that a vegetable education program would positively affect mediating factors of vegetable consumption of primary school students. We further hypothesized that a high-intensity teacher training would be more effective than a low-intensity teacher training preceding the vegetable education program on mediating factors associated with vegetable consumption amongst primary school students.

## 2. Materials and Methods

### 2.1. Study Design

A three-arm cluster-RCT was conducted to test the effectiveness of a vegetable education program preceded by two different levels of teacher training compared with a control to positively change mediating factors associated with vegetable consumption in primary school children. A cluster-RCT was undertaken with school and teacher as cluster levels because the program was delivered by the teacher and, as such, an intervention at an individual student level was not possible.

Data were collected from students at three time points: (1) at baseline (pre-test), (2) immediately after the intervention (post-test), and (3) at three-month follow-up (follow-up).

#### 2.1.1. Intervention

Two intervention groups and one wait-listed control group were included in the study. The intervention arms were the same teacher-led school-based vegetable education program VERTICAL (Vegetable Education Resource To Increase Children’s Acceptance and Liking), preceded by differing teacher training intensity, and hence, implementation effort. The three groups were the following:

1. Intervention low: VERTICAL vegetable education intervention with low-intensity teacher training (online and written materials).
2. Intervention high: VERTICAL vegetable education intervention with high-intensity teacher training (as for ‘intervention low’ but with face-to-face training of teachers).
3. Control: regular school curriculum (with VERTICAL training and materials provided post-study).

##### Teacher Training

Two forms of training to prepare teachers to teach the vegetable education intervention were evaluated, low and high intensity: (1) Low-intensity training: teachers were provided with lesson materials, a written implementation manual, as well as an online training module taking around 20 min to complete (see also Appendix A). (2) High-intensity training: teachers were provided with the same materials as in the low-intensity training (including the online training module), but additional interactive face-to-face (F2F) training (45 min) was provided by research staff involved in the study. F2F training delivered information on the same elements as delivered through the written and online materials. In addition, school-specific implementation plans were discussed with staff and a hands-on exercise, illustrative of the program, was conducted with teachers.

##### Vegetable Education Program

The classroom intervention in both intervention arms was a teacher-led vegetable education program VERTICAL designed to positively prime children towards vegetable consumption by increasing enjoyment and willingness to consume vegetables. A pilot version of the program is described elsewhere [33] and results from a student and teacher evaluation were used to further develop the program [33,34]. A description of VERTICAL can be found in Appendix A.

Classroom teachers in the two treatment arms received their allocated training and subsequently implemented the vegetable education intervention in their classrooms over a period of 5 weeks. During that time, control schools continued to teach their regular curriculum to their students. The waitlisted control schools were offered the intervention materials after completion of the study. Baseline measures for all participating children were collected at commencement of the school term (week 1 or 2) and post-test data towards the end of the same term (week 8 or 9). A follow-up measurement was conducted at three-month follow-up to determine if any effects were sustained.

#### 2.1.2. Participants and Recruitment

Participants were primary school students from Sydney, New South Wales (NSW) and Adelaide, South Australia (SA), Australia. Students were eligible if they were in year 2 to year 6 (7–12-year-old children) from a participating classroom of a participating primary school and their parent/carer had provided written consent. Recruitment of students was a staged approach, whereby schools were first approached to take part in the study, and then consent was sought from (parents/carer of) participants via participating teachers. Inclusion criteria for participating schools were: (1) government (public) primary school, and (2) school located in one of ten selected areas in Greater Sydney or Greater Adelaide. Schools were excluded from participation if they could not accommodate computerized data collection or had been previously involved in trialing the vegetable education program [34]. There were no specific eligibility criteria for teachers other than willingness to take part. Schools were requested to take part by involving a minimum of six classes (two for lower (year 2), middle (year 3–4) and upper (year 5–6) stage, respectively); however, this was not strictly enforced for practical recruitment considerations.

A stratified approach was followed to recruit schools stratified for socio-economic status (SES) in order to control for any moderating effects of SES on intervention effectiveness. Scores for Index of Relative Socio-Economic Advantage and Disadvantage (IRSEAD) were obtained from the Statistical Local Areas (SLA) of Greater Sydney (NSW) and Greater Local Areas (GLA) in Greater Adelaide (SA) [35]. Tertile splits in each state were conducted to divide areas according to low (IRSEAD deciles 1–5), medium (IRSEAD deciles 6–8) and high (IRSEAD 9–10) socio-economic status. Areas with fewer than 6 schools were excluded. A total of 4, 3 and 3 (NSW) and 3, 3 and 4 (SA) areas with low-, medium- and high-SES, respectively, were randomly selected from a list of all areas by an independent statistician using the ‘random number’ function in Excel (Microsoft Office). A list of government primary schools from each selected area was compiled using information from the Department of Education and all schools were invited to take part. Schools received a letter and follow-up contact to those that did not respond were made by phone. Once schools were recruited into the study, they were randomly allocated to one of the three treatments (two intervention or control). Allocation to treatment was done by the principal investigator in the order of enrolment date in the study using a randomized block design for each state (SES levels were blocks) created by an independent statistician prior to recruitment in each state using R (version 3.4.2, using ‘block rand’ library v1.3).

Treatment allocation was at the cluster (school/classroom) level and there was no allocation concealment at the cluster level. Written informed consent was obtained from parents for students after treatment allocation. The information sheet to parents was generically phrased as a ‘study to investigate the effect of education activities on factors related to children’s liking and consumption of foods, and specifically of vegetables’. In addition, they received one of two accompanying letters from the school. Parents in intervention schools (low- and high-intensity training) received a letter stating that their classroom would be implementing a new education program that involved hands-on tastings, including of vegetables. It mentioned that this program would be scientifically evaluated, and that their consent was required in order for their child’s data to be collected. Parents in control schools received a letter that simply repeated the information in the information letter (without reference to any intervention). This procedure was followed for two reasons: (1) To limit bias amongst students or parents as much as possible, (2) parental consent was only requested for taking part in the survey, as the decision to take part in the study was taken at the school and classroom level, and therefore, all students in participating intervention classes received the vegetable education program, regardless of whether or not their parent had provided consent for their child to take part in the survey.

Ethical approval for this study was provided by the CSIRO Human Research Ethics Committee (HREC24/2016), the NSW Department of Education and Communities (SERAP2017036) and the SA Department for Education (2018-0032). This trial was registered with the Australian New Zealand Clinical Trials Registry (ACTRN12620000392965).

### 2.2. Outcome Measures

All outcome measures were measured at the individual (student) level.

#### 2.2.1. Primary Outcome Measures

Primary outcome measures were collected by students self-completing an online questionnaire in the classroom. The survey was administered using the SurveyGizmo website. Six primary outcome measures were collected (Table 1):

- Knowledge: knowledge was tested in relation to vegetables and the senses involved in eating and drinking. A combination of multiple-choice questions, true/false statements and open questions was used.
- Verbalization: ability to verbalize sensory perceptions was tested. Children were asked to provide descriptive words for two vegetables.
- Acceptance: acceptance for vegetables was measured as a single item using an age-appropriate 7-point hedonic facial scale [36]. In addition, acceptance for six specific vegetables, which varied between year levels, was measured using the same scale. Examples to ensure correct understanding of the scale were given.
- Behavioural intention: behavioural intentions for eating a variety of foods and vegetables was measured using four statements and 5-point Likert scales. Format and response categories were according to the validated scales of behavioural intent from the Theory of Planned Behaviour [37].
- Willingness to try: willingness to try (yes/no) four specific (less commonly consumed) vegetables was measured using pictures of the vegetables.
- Number of new vegetables tried: students were asked to record the number of new vegetables they had tried in the previous month.

Outcome measures were the same for all students, but three different versions of the survey were used for different year levels (i.e., lower, middle and upper) to correspond with the resource content of each unit. Differences between survey versions related to the knowledge questions and the specific vegetables used in the verbalization, acceptance and willingness to try questions. The questionnaire for the lowest year level had no open questions in the knowledge component to accommodate the limited writing capabilities and shorter attention span of this younger age-group.

To assist with comprehension and task requirements, teachers explained the task of completing the survey questionnaire to the children. Year 2 students completed the questionnaire through class-guided support from the teacher (the teacher read out the questions and/or showed on the interactive white board). Students in year 3–6 self-completed the questionnaire at their own pace, with the teacher present to answer any questions. The survey questionnaire took a maximum of 15 min to complete and was essentially a shorter version of a previously used questionnaire [34].

#### 2.2.2. Secondary Outcomes and Other Measures

At baseline, information from students was collected on age and gender. Information from parents (embedded in the consent form) at baseline was collected on their child’s cultural background (using categories of the Australian Bureau of Statistics) [38], as well as level of food neophobia using a validated scale [39] and vegetable consumption using a child-adapted version [40] of a validated scale for Australian adults [41].

In the consent form, parents were asked if they consented to being contacted for follow-up about this study. This aimed to collect information on child food neophobia and vegetable consumption after the intervention, for use as secondary measure (comparison with baseline). However, only 12.5% of parents (*n* = 205) completed the follow-up survey. The small sample size meant the study was underpowered for secondary outcome measures and these were, therefore, not analyzed.

### 2.3. Statistical Analyses

#### 2.3.1. Sample Size Calculation/Power

The study was a cluster-RCT, using a stratified design, whereby schools were randomly allocated to treatment, and all individuals in participating classes within that school were subjected to the same treatment. As there is dependence between individuals sampled from the same school and classroom, the clustered nature was accounted for in the power analysis.

Sample size was calculated based on a change in student behavioural intention outcomes with a small effect size of 0.15 based on comparable studies [32,34]. With a power of 0.95, alpha = 0.05 and using a repeated exposure ANOVA with 3 treatment groups, an overall sample size of 531 students was needed (GPower 3.1.9.2). This sample size was multiplied by a correction factor of 1 + (m − 1) ρ, called the design effect (where m is the average cluster size and ρ is the intra-class correlation coefficient), to take the clustered nature of the data into account [42]. Assuming an m of 25 students per class and estimating a small degree of correlation (ρ = 0.05), the correction factor is 2.2. Thus, a minimum sample of 1168 students was needed (531 × 2.2 = 1168). On a cluster level, we sought to obtain data from 30 schools (10 schools in each of the three treatment arms) to cover a wide geographic and socio-economic spread. With an estimated response rate in classes of around 30% [43], and a minimum participation of 6 classes per school, we expected to receive data from a minimum of 1350 students (30 schools × 6 classes × 25 students/class × 30% response rate).

#### 2.3.2. Data Coding

Before proceeding to data analysis, data were treated as follows:

- Knowledge: A sum score was calculated. A total of 11 points for knowledge could be scored. For all questions, a correct response provided a score of 1 point, with exception of an open question about listing vegetables, where up to 2 points could be scored (year 3–4: 0 correct = 0 points, 1–3 correct = 1 points, 4 or more correct is 2 points; year 5–6: 0 correct = 0 points, 1 correct = 1 point, 2 or more correct = 2 points). Cut-offs were determined based on the results from a pilot study [34].
- Verbalization: The number of descriptive (e.g., crunchy, sweet) words was counted. Hedonic words (e.g., delicious, yummy) were excluded. One point was allocated for each correct answer. The number of descriptive terms summed across the two vegetables was calculated.
- Acceptance: Cronbach’s alpha was calculated to determine internal consistency of individual items to the overall concept and was satisfactory (0.75). An average score across all items was calculated.
- Behavioural intention: Cronbach’s alpha was calculated and was satisfactory (0.80). The mean of these items was calculated.
- Willingness to try: A sum score was calculated with one point allocated for each vegetable the child was willing to try.
- Number of new vegetables tried: the number of new vegetables the student recorded.

#### 2.3.3. Statistical Data Analysis

Participants were included in the analysis of the intervention effect when they had completed baseline and at least one of the two post-intervention surveys. Other participants were considered to be drop-outs. Participant characteristics of the sample for analysis and drop-outs were compared using Pearson Chi-Square Analysis.

To statistically analyze whether there were differences between the low- and high-intensity training preceding the vegetable education intervention, mixed linear modelling (MLM) was conducted. Analyses were conducted on outcome measures with time point (baseline, post-test, follow-up), treatment condition (intervention low, intervention high and control), year level (lower, middle, upper) plus the 2- and 3-way interactions between time point, treatment condition and year levels as fixed factors. The low-intensity intervention arm was set as the contrast category, and it was determined whether the high-intensity intervention significantly differed from the low intensity. This multi-level statistical analysis took the interdependency of measures into account (i.e., students over time, students in the same class, classes in the same school). Gender, school size, socio-economic status of the school’s area and state were included in the models as covariates.

Where there was no statistically significant difference between the two different intensity levels of training preceding the vegetable education intervention at post-test, these two groups were combined to analyze the effect of the vegetable education program using mixed linear modelling (MLM). These statistical analyses were conducted on outcome measures with time point (baseline, post-test, follow-up), treatment condition (both intervention groups combined vs control group), year level (lower, middle, upper) plus the 2- and 3-way interactions between time point, treatment condition and year levels as fixed factors. The 2-way interaction effect between treatment condition and time point was used to interpret the effects of teaching the vegetable education program over time, and the 3-way interaction to see if this varied by year level. Multi-level interdependency and covariates were defined as described in the first step of the analyses.

All data analyses were performed using Stata v15 (www.stata.com). Statistical significance was set at *p* < 0.05.

## 3. Results

### 3.1. Participants

**Figure 1:**
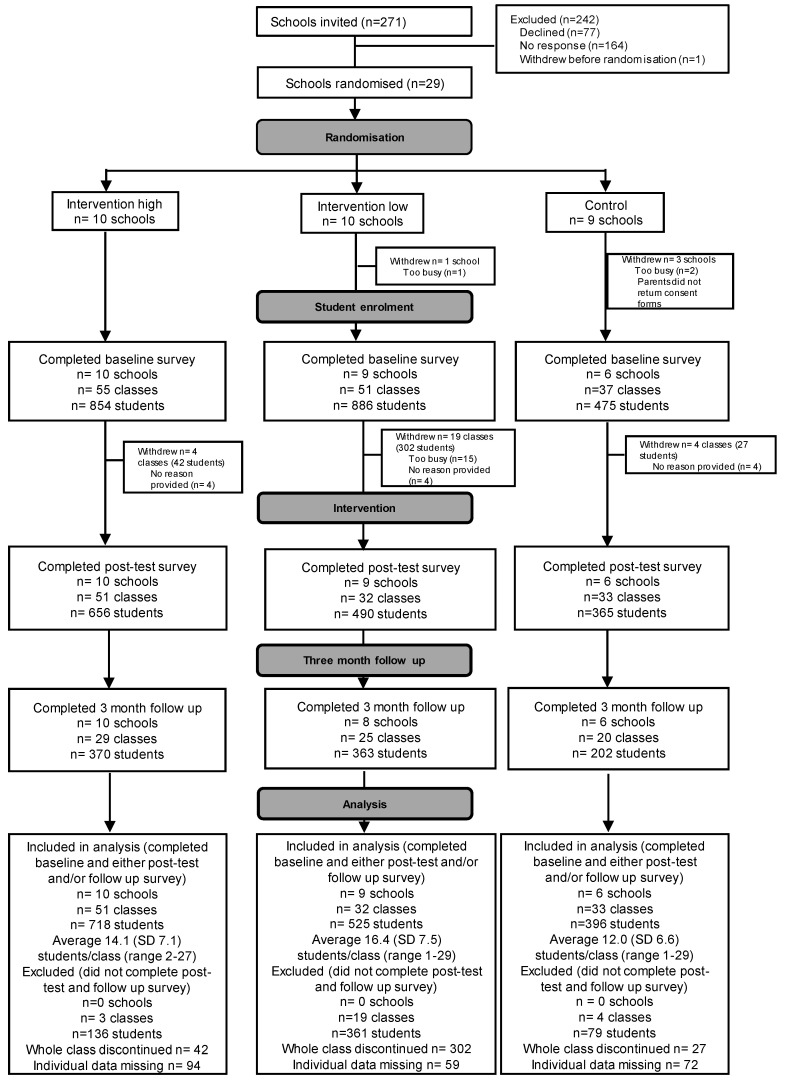
CONSORT participation flowchart of schools, classes and students in NSW and SA.

Recruitment was conducted from September 2017 to February 2018 in NSW, with the study conducted between April and October 2018. In SA, the recruitment period was April–June 2018 and the study took part July–December 2018. A total of 25 schools took part in the intervention study. There were 1639 students from 116 classes who completed baseline data plus the post-test and/or three-month follow-up test (Figure 1). There were a larger number of schools from the control arm who withdrew after they were allocated (3 out of 9).

A total of 2215 students completed the baseline test and 576 students of those (26%) did not complete further assessments. These drop-outs were largely (64% of participants) due to whole classes not continuing, particularly in one school in the low-intensity intervention arm who initially intended to take part with the whole school but decided to continue with a selected number of classes after the baseline survey due to time constraints. As a result, there was a difference in the drop-out rate by intervention arm (Supplementary Material).

Students who did not continue after baseline measurements (“drop-outs”) did not significantly differ from students who remained in the trial in terms of year level or gender (Supplementary Material). However, they differed in SES of the suburb in which their school was located, the state they lived in and school size. Drop-outs were more likely to come from schools located in medium SES areas, schools located in South Australia and from medium-sized schools.

Table 2 shows that the random allocation resulted in participants in the three different arms being similar in most characteristics, but there was an imbalance in SES of the suburb in which their school was located and school size. A relatively larger proportion of students in the control group were from schools located in high-SES areas. Further, more schools in the high-intensity training intervention arm were relatively smaller (<400 students) than the other two arms. These factors were co-variates in the MLM analysis thereby controlling for any effect they may have on outcome measures.

### 3.2. Outcome Measures

The comparisons between the low- and high-intensity training intervention groups did not yield any significant differences on any of the outcomes at post-test, meaning that student outcomes were not affected by the intensity of training the teacher had prior to teaching the vegetable education (Table 3).

**Figure 2:**
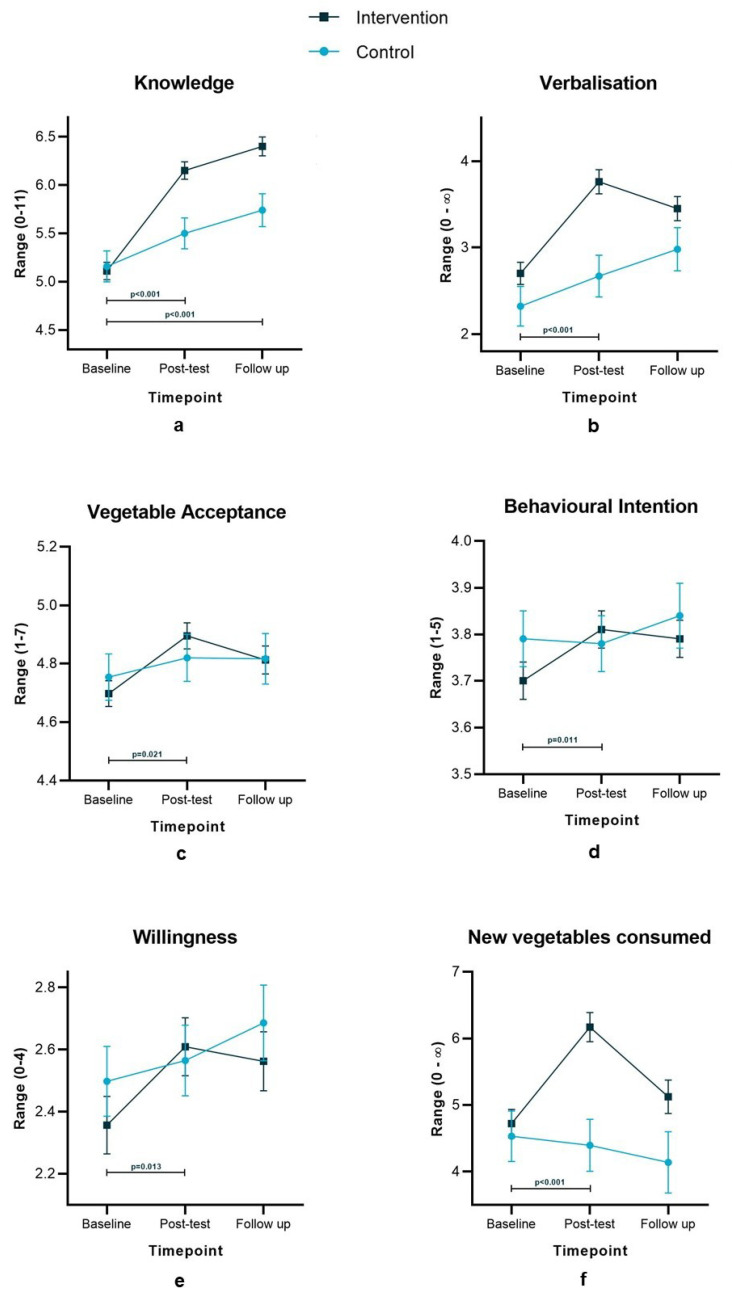
Changes in outcomes over time in both intervention groups combined compared to the control group in (**a**) knowledge about vegetables and the senses, (**b**) ability to verbalize sensations, (**c**) vegetable acceptance, (**d**) behavioural intentions, (**e**) willingness to eat vegetables, (**f**) number of new vegetables consumed. *p*-value on the difference in intervention * time point interaction effect between the intervention and control.

The lack of significant differences between low- and high-intensity training preceding the intervention meant both intervention arms could be combined for all outcome measures. Results are presented in Figure 2. Looking at the change from baseline to post-test (Table 4), the MLM analyses showed significant positive effects of the vegetable education intervention compared to the control group on all six outcomes: knowledge (*p* < 0.001), verbalization skills (*p* < 0.001), behavioural intention (*p* = 0.011), willingness to try (*p* = 0.013), vegetable acceptance (*p* = 0.021), and new vegetables consumed (*p* < 0.001). At the three-month follow-up, this was only sustained for knowledge (*p* < 0.001).

There were no significant covariates, two-way interactions other than the intervention by time effect, or three-way interactions. This meant that the effect of the intervention was independent of year level, gender, socio economic status, school size or state.

## 4. Discussion

This cluster-RCT showed that a short (5 h) teacher-led vegetable education program was effective in changing mediating factors associated with vegetable consumption among primary school students. This effect was independent of whether teachers had received additional face-to-face training or not prior to teaching the program to their students.

The VERTICAL program increased student’s vegetable knowledge, ability to verbalize sensations when eating vegetables, vegetable acceptance, behavioural intention to eat, willingness to try them, as well as the number of new vegetables consumed. A matched-controls pilot study on an earlier version of the VERTICAL program similarly found statistically significant increases in knowledge, ability to verbalize sensations, vegetable acceptance and vegetables willing to try [34]. The current study found a statistically significant increase in behavioural intentions to eat vegetables, whereas the previous study did not [34]. This might be due to the increased power of the current study and/or the slight improvements in the intervention materials. The current study also found an increase in the number of new vegetables consumed, whereas the previous study did not find an increase in vegetables tried [34]. This means that the current program demonstrated positive effects on actual behavioural measures of consumption, whereas the pilot version did not. However, as question formats differed slightly, results cannot be directly compared.

The current intervention is a short (5 × 1 h) intervention, which is much shorter than most classroom-based experiential learning programs aimed to support vegetable consumption and/or healthy eating, that typically involve 10–18 h [25,28,29,30,31,32]. These, mostly European, programs try to influence vegetable consumption and associated mediating factors either directly [25,44] or indirectly [28,29,30,31,32] using taste lessons with or without other components such as peer-modelling [25] or gardening [44], whilst there are also interventions focusing on school vegetable provisioning (e.g., [45]) or multi-component fruit-and-vegetable interventions where education is only one element [46]. Focusing on studies most comparable to the VERTICAL intervention in terms of scope and outcome measures, the VERTICAL intervention affected more student outcomes than a Belgian vegetable-specific program using classroom tastings and gardening [44], whereas it was similar in outcomes to an Italian fruit-and-vegetable program focused around repeated exposure, reward and peer modeling of longer duration [25]. A Dutch sensory education program focused on healthy eating with similar duration (5 × 45 min) was effective in increasing knowledge and tasting familiar vegetables but showed no changes in behavioural intention, willingness to try vegetables and tasting unfamiliar vegetables [26,27]. Thus, the VERTICAL intervention achieved positive change in more factors using a comparable intervention duration. The difference between the two programs is that the theoretical framework of VERTICAL is specifically derived from insights on development of vegetable acceptance in children and focuses more on affective and implicit components.

The effect of the vegetable education program was sustained at three-month follow-up for knowledge and not sustained for the other outcome measures. There are not many classroom-based experiential learning programs aimed to support healthy eating that have included follow-up measurements, but the current study mimics available results in that sustained effects were found for knowledge [32] and not for other comparable outcome measures [25,28,31,32]. According to behaviour change theories, cognitions are easier to sustainably change than attitudinal and behavioural components [37]. Therefore, multiple reinforcements are likely necessary for sustained change. The program consists of three units throughout primary school and thus could provide such reinforcement when students are taught the vegetable education program throughout their primary school career. However, any cumulative effect has not been investigated and, therefore, outcomes remain speculative.

There was no difference in student outcomes between students whose teachers had received online and written training alone and teachers who had received additional face-to-face training. The two different levels of intensity of teacher training differ greatly in their cost structure. The low-intensity training consists primarily of one-off costs related to development of materials whereas the high-intensity version consists of additional per school costs to implement the face-to-face training component. Other forms of direct teacher support with a lower cost structure would be available, for example webinars, teaching only selected teachers in schools, etc. In this study, the high-intensity training delivered the training in a way that potentially had the most impact on teachers, i.e., face-to-face contact, with the training delivered by researchers with expertise in the scientific framework that underpins the program. As there was no difference in student outcomes between these two training levels, face-to-face or other forms of direct interaction with teachers are not needed to achieve students positively benefiting from the intervention which has great benefits for the scalability of the intervention. The implementation costs of the program with a low-intensity training version are low, since all materials can be provided via one platform (e.g., website), thus offering opportunities for a national roll out at relatively low costs. Promotion and communication are essential for uptake of the program. Direct engagement with schools and teachers is likely to be the best method to promote uptake of the program, as indicated by experiences during recruitment, and the drop-out rate of those that were assigned to the control arm being higher than the intervention arms.

The potential benefits of this program include increased vegetable consumption. No adverse events were reported. The aim of the program is behaviour change; however, all outcomes are achieved through curriculum aligned activities, which means that it does not take away precious time from key learning outcomes.

There are several limitations of the current study that need acknowledgement. There was a dropout bias by intervention group, as more schools allocated to the wait-listed control arm withdrew their participation than schools allocated to the intervention arms. It was clear during recruitment that all schools that agreed to participate were motivated to implement the vegetable education program. The reason for withdrawal provided by two schools was being too busy, but it seemed that disappointment with being allocated control school was the primary motivation to withdraw. There was also one control school where no parents returned the consent form. This school was located in a low SES area (9th decile of disadvantage) and school staff indicated that low literacy and English language comprehension skills meant parents had difficulty comprehending the information sheet and consent form causing this non-response. This demonstrates the ethical dilemma between the ethical requirements of informed consent and the need to include all levels of SES in interventions to develop equitable programs that support closing the health inequitably gap between low- and high-SES populations. It would be recommended to consider much more simplified versions of informed consent forms for groups with low literacy levels and English skills and/or consent forms in other familiar languages used.

Control schools that withdrew were from low (n = 2) and medium (n = 1) SES. The resulting effect is that the control group had a higher proportion of participants from high-SES than the intervention arms. This variable was included as covariate in the mixed model analysis, thereby reducing its effect. However, if students from a higher SES background learn quicker [47], this means that the effects of the vegetable education program may be underreported. Another limitation is that the same questionnaire was used at baseline and follow-up measures. This was done to ensure that scores could be directly compared, as it is very difficult to design different questionnaires that would yield exactly the same results in the same conditions. However, it might have led to a learning effect, in particular relevant to the more cognitive outcome measures, knowledge and verbalization skills. This is supported by the continual increases in these measures in the control group, and may have contributed to verbalization skills not attaining a sustained intervention effect. There was a low response rate for the secondary measurements collected from parents. This meant the effect of the intervention on the child’s vegetable consumption could not be established. In future research, further strategies to increase retention rates would be recommended or (with training) data may be collected from students themselves.

This study represents a good external validity for the Australian context as research was undertaken in two different states and in all areas of socio-economic status. The results show that there is a strong evidence base for efficacy of the program, regardless of student’s backgrounds in terms of gender, year level, school size, socio-economic status and state of residence. Three different units with slightly different content were developed for different year levels. The lack of significant effects with year level shows that all units performed equally well. The two youngest years in primary school (foundation and year 1) were not included in this study due to the young age of these children prohibiting them from taking part in a written survey. Thus, the effectiveness of the vegetable education program for these groups remains to be evaluated.

Acceptance is the key barrier to vegetable consumption by children in most Western countries. As such, the same scientific framework can be applied in education programs for other Western countries and other countries where low acceptance is limiting intake. However, for this intervention, the structure and specific activities were based on the Australian curriculum and Australian school context to facilitate uptake by teachers, and it would be recommended to use a similar approach when adapting the program to other countries.

The current program has a solid evidence base. Therefore, national roll-out of this program amongst Australian primary schools has the potential to increase vegetable acceptance and consumption of Australian children on a large scale, thereby helping establish lifelong healthy eating habits.

## Figures and Tables

**Table 1:** Primary outcome variables (Cronbach α), number and example of question format and answer category.

Outcome	Number of Questions	Example of Question	Answer Category
Knowledge	11	Which 5 senses are involved in eating vegetables?	Multiple Choice, True/False, Open question
Verbalization ability	2	How does this [vegetable] taste and feel in our mouth? Write as many describing words as you can.	Open question
Vegetable acceptance (0.75)	7	How much do you like [vegetable]?	From ‘Really dislike’ (=1) to ‘Really like’ (=7)
Behavioural intention (0.80)	4	I will eat a variety of vegetables.	From ‘No, definitely not’ (=1) to ‘Yes, definitely’ (=5)
Vegetables willing to try	4	Would you try [vegetable] if someone offered it to you?	Yes/No
New vegetables consumed	1	How many new vegetables have you consumed in the last month?	Number

**Table 2:** Baseline characteristics of the individual student participant level by randomized group, high-intensity teacher training followed by vegetable education (intervention high), low-intensity teacher training followed by vegetable education (intervention low), or regular classroom education (control).

Characteristics	Intervention High (*n* = 718)	Intervention Low (*n* = 526)	Control (*n* = 396)
Age, mean (SD), years	8.99 (1.53)	9.18 (1.38)	9.23 (1.43)
Gender (%)			
Boy	332 (47.2)	258 (49.1)	205 (51.8)
Girl	386 (53.8)	267 (50.9)	191 (48.2)
Cultural background ^1^			
Australian/New Zealander	281 (53.4)	321 (71.0)	197 (65.9)
Northern/Western European	57 (10.8)	43 (9.5)	40 (13.4)
Southern/Eastern European	31 (5.9)	29 (6.4)	13 (4.3)
North African/Middle Eastern	18 (3.4)	2 (0.4)	7 (2.3)
South East Asian	35 (6.7)	13 (2.9)	5 (1.7)
North East Asian	19 (3.6)	17 (3.8)	13 (4.3)
Southern/Central Asian	51 (9.7)	12 (2.7)	10 (3.3)
North/Central/South American	13 (2.7)	10 (2.2)	2 (0.7)
Sub Saharan African	5 (1.0)	0 (0)	4 (1.3)
Other (not specified)	16 (3.0)	5 (1.1)	8 (0.7)
Vegetable consumption, mean (SD) serves/day ^1^	1.63 (1.14)	1.71 (1.16)	1.79 (1.20)
Food neophobia, mean (SD) ^1^	14.30 (4.64)	14.16 (4.69)	13.70 (4.75)
Year level ^2^			
Lower	193 (26.9)	77 (14.7)	87 (22.0)
Middle	300 (41.8)	273 (52.0)	152 (38.4)
Upper	225 (31.3)	175 (33.3)	157 (39.6)
SES ^3^			
Low	211 (29.4)	233 (44.4)	52 (13.1)
Medium	366 (51.0)	173 (33.0)	98 (24.7)
High	141 (19.6)	119 (22.7)	246 (62.1)
State			
NSW	273 (38.0)	189 (36.0)	166 (41.9)
SA	445 (62.0)	336 (64.0)	230 (58.1)
School size			
<400 students	545 (75.9)	322 (61.3)	90 (22.7)
401–600 students	173 (24.1)	84 (16.0)	196 (49.5)
>600 students	0 (0)	119 (22.7)	110 (27.8)

Values are numbers (percentages) unless stated otherwise. ^1^ Data collected from parents, available for 1277 (77.9%), 1269 (77.4%) and 1288 (78.6%) students for cultural background, vegetable consumption and food neophobia respectively. ^2^ Year level: lower = year 2, middle = year 3 and 4, upper = year 5 and 6. ^3^ Based on Index of Relative Socio-Economic Advantage and Disadvantage (IRSEAD) scores from Australian Bureau of Statistics, Low = IRSEAD deciles 1–5, medium = IRSEAD deciles 6–8, high = IRSEAD deciles 9–10.

**Table 3:** Difference between low- and high-intensity teacher training preceding teaching the vegetable education program on student outcomes over time.

Outcome	*n*	Effect (95% CI)	Bonferroni *p*	ICC Class/School
Knowledge	1627			0.109/0.000
Baseline to Post-test		−0.194 (−0.465 to 0.077)	0.296	
Baseline to Follow-up		−0.243 (−0.566 to 0.081)	0.243	
Verbalization	1639			0.077/0.008
Baseline to Post-test		−0.055 (−0.376 to 0.265)	1.000	
Baseline to Follow-up		0.003 (−0.382 to 0.387)	1.000	
Vegetable acceptance	1622			0.023/0.000
Baseline to Post-test		−0.047 (−0.178 to 0.084)	1.000	
Baseline to Follow-up		−0.113 (−0.269 to 0.043)	0.284	
Behavioural intention	1621			0.012/0.000
Baseline to Post-test		−0.075 (−0.191 to 0.041)	0.425	
Baseline to Follow-up		−0.139 (−0.276 to −0.001)	0.046 *	
Vegetables willing to try	1621			0.011/0.000
Baseline to Post-test		0.088 (−0.085 to 0.262)	0.811	
Baseline to Follow-up		−0.038 (−0.243 to 0.167)	1.000	
New vegetables consumed	1612			0.030/0.001
Baseline to Post-test		−0.315 (−1.311 to 0.681)	1.000	
Baseline to Follow-up		0.049 (−1.128 to 1.225)	1.000	

Note: positive and negative effects indicate changes from baseline. * *p* < 0.05.

**Table 4:** Difference in student outcomes over time between students who have received the vegetable education regardless of intensity of teacher training (intervention) and students who followed their regular curriculum (control).

Outcome	*n*	Effect (95% CI)	Bonferroni *p*	ICC Class/School
Knowledge	1627			0.113/0.000
Baseline to Post-test		0.724 (0.482 to 0.966)	<0.001 *	
Baseline to Follow-up		0.732 (0.432 to 1.033)	<0.001 *	
Verbalization	1639			0.086/0.022
Baseline to Post-test		0.709 (0.420 to 0.998)	<0.001 *	
Baseline to Follow-up		0.082 (−0.276 to 0.440)	1.000	
Vegetable acceptance	1622			0.030/0.000
Baseline to Post-test		0.132 (0.016 to 0.248)	0.021 *	
Baseline to Follow-up		0.053 (−0.092 to 0.197)	0.823	
Behavioural intention	1621			0.018/0.000
Baseline to Post-test		0.126 (0.024 to 0.229)	0.011 *	
Baseline to Follow-up		0.044 (−0.084 to 0.171)	0.884	
Vegetables willing to try	1621			0.015/0.000
Baseline to Post-test		0.186 (0.033 to 0.338)	0.013 *	
Baseline to Follow-up		0.018 (−0.171 to 0.207)	1.000	
New vegetables consumed	1612			0.032/0.000
Baseline to Post-test		1.589 (0.709 to 2.469)	<0.001 *	
Baseline to Follow-up		0.797 (−0.291 to 1.886)	0.201	

Note: positive effects indicate increases in the difference between intervention and control groups. * *p* < 0.05.

## Supplementary Materials

The following are available online at https://www.mdpi.com/2072-6643/12/8/2343/s1, Table S1. Characteristics of study participants in the total sample, the students who remained in the trial and drop-out after baseline.

## Appendix A. Description of VERTICAL Program and Materials

The VERTICAL program was developed by sensory and consumer scientists, behavioural nutritionists and educators and aimed to meet three main objectives: (1) to be effective in achieving change amongst children in factors known to be associated positively with vegetable consumption; (2) fulfilling curriculum objectives, and (3) facilitate ease of use by teachers in the classroom. The intervention is a short intervention consisting of 5 one-hour lessons. The theoretical framework is based on scientific evidence on development of vegetable acceptance in children, with critical elements being exposure, tastings, fun, creating a positive peer-modelling culture and building affect. This science framework is combined with elements of sensory education programs, in particular students are taught how senses are involved in eating foods and they learn and apply a protocol for tasting vegetables using objective, descriptive words. Children are taught, explicitly and through exercises, that their food preferences can change with repeated eating and are encouraged to become a ‘food adventurer’. Vegetables are tasted in each lesson. Specific vegetables are recommended in each lesson that fit the lesson purpose and alternatives are included. If vegetable recommendations are followed, students taste a minimum of 10 different vegetables in each unit. The program has a strong affective component and as most lesson activities have tastings built in as part of a larger objective, implicit learning is a major pathway to developing acceptance. The cognitive component of the program explicitly steers away from cognitive reasoning that vegetables should be consumed because of their health benefits, as this has been shown to undermine acceptance [48,49]. Lesson plans are curriculum aligned and include various experiential learning activities, such as science experiments on the taste of vegetables, cultural diversity, regionality and seasonality of vegetables, growing and preparing vegetables, and processing of vegetables (Table A1). The last lesson consists of a meal with vegetables prepared and enjoyed by students together, promoting conviviality and commensality.

The program uses the BSCS 5E Instructional model throughout the five lessons; Engage, Explore, Explain, Elaborate, Evaluate [50]. There are three different units (integrated lesson plans) for three different stages of primary school: lower (5–8 years), middle (8–10 years) and upper (10–12 years). Units build onto each other, with several topics (such as the senses) recurring and building in content complexity. The resource is cross-curricular, and particularly aligns to the key learning areas of English, Science, Mathematics and Physical Health and Education, as well a generic capabilities (such as creative and critical thinking) and cross-curricular priorities (such as sustainability).

The materials of the VERTICAL program consist of the following:

- Fully written lesson plans with objectives, materials needed and suggested lesson activities. Lessons contain student worksheets and are supported by interactive whiteboard materials.
- An online teacher training module with information on program objectives and structure, theoretical background about the senses and food preference development and practical information to implement the program in the school and information.
- An implementation manual which contains the information in the training module in some more detail, and additionally provides resources for implementation of the program (e.g., shopping lists) and information on curriculum alignment.

**Table A1:** Outline of curricula lesson topics of Vegetable Education Resource To Increase Children’s Acceptance and Liking.

Number	Title	Main Topic Studied
Lower (Foundation–Year 2)		
1	The Five Senses	Students learn about the senses involved in eating and describe vegetables in terms of the five senses.
2	From Seed to Vegetable	Students discover and eat different parts of vegetable plants.
3	The Basic Tastes	Students can recognise the four basic tastes and can identify the dominant taste in different vegetables.
4	Becoming a Food Adventurer	Students learn that liking of foods can change by trying and become more open to taste novel foods.
5	Picnic in Class: Sandwich	Students prepare and enjoy eating a sandwich with vegetables together.
Middle (Year 3–4)		
1	Discover Vegetables through the Senses	Students become aware of individual differences in vegetable preferences through tasting vegetables.
2	Vegetables Grow in Different Climates	Students discover and eat vegetables from different climatic regions.
3	Preparing Vegetables—a Science Experiment	Students investigate the role of cooking on taste/texture of vegetables through a simple science experiment.
4	Perfectly Imperfect Vegetables	Student learn how visual cues can affect our food choices and try to convince someone to try an imperfect vegetable.
5	MasterChef^®^ in Class: the Salad	Students prepare, evaluate and enjoy eating a salad with vegetables together.
Upper (Year 5–6)		
1	How our Senses Interact	Students discover how our senses interact when we eat foods.
2	A Science Experiment on Taste of Vegetables	Students understand the different elements of a scientific investigation by planning and conducting an experiment on the taste of vegetables.
3	Vegetables from Farm to Plate	Students investigate the role of food technology in producing vegetable products.
4	Vegetables and Cultural Diversity	Students understand how cultural background shapes food preferences from an early age.
5	The Vegetable Dip Challenge	Students prepare, evaluate and enjoy eating vegetable dips together.
