# Supplementary material for: Effect of Experiential Vegetable Education Program on Mediating Factors of Vegetable Consumption in Australian Primary School Students: A Cluster-Randomized Controlled Trial
Source: Nutrients. 2020 Aug 5;12(8):2343. doi: 10.3390/nu12082343 (PMC7468916; doi:10.3390/nu12082343)
Supplement: Supplementary file 1 [file nutrients-12-02343-s001.pdf]

nutrients

MDPI

Supplementary data

Table S1. Characteristics of study participants in the total sample, the students who remained in the trial and drop-out after baseline.

Characteristics	Total at baseline (*n* = 2215)	Students in analysis (*n* = 1639)	Student drop-outs (*n* = 576)	*p* Value [1]
Intervention (%)				
Intervention low	40.0%	32.0%	62.7%	
Intervention high	38.5%	43.8%	23.6%	<0.0001
Control	21.4%	24.2%	13.7%	
Year level [2] (%)				0.538
Lower	22.3%	21.8%	24.0%	
Middle	44.1%	44.2%	43.6%	
Upper	33.6%	34.0%	32.5%	
Gender (%)				0.741
Boy	48.7%	48.5%	49.3%	
Girl	51.3%	51.5%	50.7%	
SES [3] (%)				<0.0001
Low	28.9%	30.3%	25.0%	
Medium	44.8%	38.9%	61.8%	
High	26.3%	30.9%	13.2%	
State (%)				0.002
NSW	36.4%	38.3%	31.1%	
SA	63.6%	61.7%	68.9%	
School size (%)				<0.0001
<400 students	50.9%	58.4%	29.5%	
401-600 students	38.1%	27.6%	67.9%	
>600 students	11.0%	14.0%	2.6%	

[1] *p* value based on Pearson Chi-Square analysis
[2] Year level: lower = year 2, middle = year 3 and 4, upper = year 5 and 6
[3] Based on Index of Relative Socio-Economic Advantage and Disadvantage (IRSEAD) scores from Australian Bureau of Statistics, Low = IRSEAD deciles 1-5, medium = IRSEAD deciles 6-8, high = IRSEAD deciles 9-10.
